# Run-on of germline apoptosis promotes gonad senescence in *C. elegans*

**DOI:** 10.18632/oncotarget.9681

**Published:** 2016-05-31

**Authors:** Yila de la Guardia, Ann F. Gilliat, Josephine Hellberg, Peter Rennert, Filipe Cabreiro, David Gems

**Affiliations:** ^1^ Institute of Healthy Ageing, and Department of Genetics, Evolution and Environment, University College London, London, UK; ^2^ Department of Structural and Molecular Biology, University College London, London, UK

**Keywords:** apoptosis, C. elegans, hyperfunction, pathology, senescence, Gerotarget

## Abstract

Aging (senescence) includes causal mechanisms (etiologies) of late-life disease, which remain poorly understood. According to the recently proposed hyperfunction theory, based on the older theory of antagonistic pleiotropy, senescent pathologies can arise from futile, post-reproductive run-on of processes that in early life promote fitness. Here we apply this idea to investigate the etiology of senescent pathologies in the reproductive system of *Caenorhabditis elegans* hermaphrodites, particularly distal gonad degeneration and disintegration. Hermaphrodite germ cells frequently undergo “physiological” (non-damage-induced) apoptosis (PA) to provision growing oocytes. Run-on of such PA is a potential cause of age-related gonad degeneration. We document the continuation of germline apoptosis in later life, and report that genetically blocking or increasing PA retards or accelerates degeneration, respectively. In wild-type males, which lack germ line apoptosis, gonad disintegration does not occur. However, mutational induction of PA in males does not lead to gonad disintegration. These results suggest that as germ-cell proliferation rate declines markedly in aging hermaphrodites (but not males), run-on of PA becomes a pathogenic mechanism that promotes gonad degeneration. This illustrates how hyperfunction, or non-adaptive run-on in later life of a process that promotes fitness in early life, can promote atrophic senescent pathology in *C. elegans*.

## INTRODUCTION

Biogerontologists traditionally use lifespan as a measure of aging. However, aging leads to death by causing the development of senescent pathologies [1, 2]. Arguably, discovering the causes (etiologies) of such pathologies is key to understanding aging. Senescence causes numerous pathologies, and which pathologies limit life varies according to species, individual and environment. Thus, in a model organism like *C. elegans*, understanding how a given senescent pathology originates is of interest, whether it limits lifespan or not. This study therefore focuses not on lifespan but on senescent pathology.

The biological mechanisms of senescence in *C. elegans* remain poorly understood. One suggested cause of aging is stochastic molecular damage, e.g. oxidative damage [3, 4]. However, difficulty in demonstrating this in *C. elegans* [5–7] has increased interest in possible alternative mechanisms. According to evolutionary theory, a major cause of aging is antagonistic pleiotropy; specifically, the deleterious effects later in life of wild-type genes that promote fitness earlier in life [8]. In terms of proximate mechanisms, antagonistic pleiotropy can manifest e.g. as physiological costs of reproduction [9, 10], or as destructive late-life gene action (run-on or *hyperfunction*) [11, 12]. Such run-on, or pathogenic continuation in later life of processes that promote fitness in early life, has been likened to a faucet left on [13, 14], and can lead to lethal senescent pathology through overgrowth (hypertrophy or hyperplasia, e.g. cardiac hypertrophy or cancer, respectively) or atrophy (e.g. osteoporosis). Such antagonistic pleiotropy-related mechanisms imply that the development of senescent pathologies can, like development *per se*, be understood as genetically determined phenotypes. Thus, the developmental genetics of senescent pathologies may be key to understanding aging.

**Figure 1:**
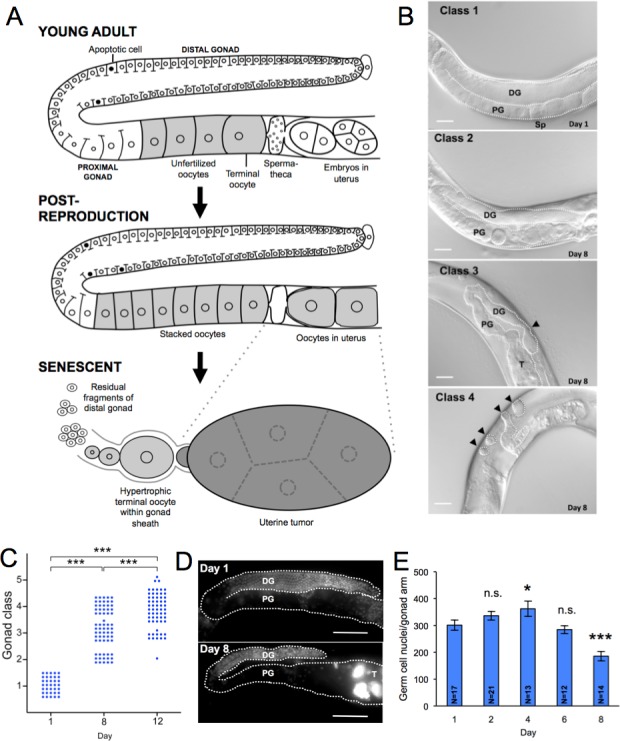
Aging pathology in the *C. elegans* hermaphrodite gonad **A.** Schematic summary of major age changes in the anatomy of the hermaphrodite gonad, derived from previous sources [16, 18–20, 62, 63] and this study. Top, young, reproductive adult. Middle, post-reproductive adult, shortly after sperm depletion; note stacking of oocytes in proximal gonad and presence of oocytes in the uterus. Bottom, terminal state after pathogenetic changes in the aging gonad. Fragments comprised of clumps of cellularized germ cells are all that remains of the distal gonad, the terminal oocyte has undergone hypertrophy, and oocytes within the uterus have developed into a large tumor. **B.** Stages 1 - 4 of gonad deterioration; images from Nomarski microscopy. Class 3 arrowhead, narrowing of distal gonad. Class 4 arrowheads, distal gonad fragments. Scale bar, 20 μm. **C.** Age increase in gonad degeneration (wild type hermaphrodite). Each dot represents an individual animal. *** *p* < 0.001, Wilcoxon-Mann Whitney test. **D.**, **E.** Numbers of germline nuclei decline with age. **D.** Gonads of young and old hermaphrodites stained with DNA-staining fluorescent dye (DAPI); scale bar, 20 μm. Note smaller number of nuclei in 8 day old worm. T, DNA mass in uterine tumor. PG, proximal gonad, DG, distal gonad. **E.** Age change in number of germline nuclei (stage 4, 5 worms on day 8 not included); * 0.01 < *p* < 0.05, *** *p* < 0.001, Student's t test, compared to day 1; error bars, S.E.M.; n.s., not statistically significant. N, sample size.

In *C. elegans* hermaphrodites, age-related pathologies suggestive of hyperfunction affect a number of organs [15]. This includes the gonad, which consists of two U-shaped gonad arms linked to a single central uterus. At the distal end of each gonad is a population of mitotic stem cells, some of which undergo meiosis, eventually forming sperm and then, subsequently, large oocytes in the proximal gonad arm (Figure 1A). Mature oocytes are then ovulated into the spermatheca where they are fertilized by sperm, and then pass *via* the uterus and vulva into the world beyond.

During aging, the hermaphrodite gonad undergoes a striking morphological transformation (Figure 1A). Initially, after depletion of sperm, unfertilized oocytes accumulate [16] and enter the uterus, where they become hypertrophic, forming large tumors [17–19]. By contrast, the aging distal gonad undergoes severe atrophy and eventual disintegration [16, 18, 20].

In this study, we take a developmental pathology approach to investigate *C. elegans* gonad senescence, and present evidence that post-reproductive physiological apoptosis (PA) in the germline contributes to distal gonad degeneration.

## RESULTS

### The aging hermaphrodite germline undergoes atrophy and hypertrophy

We first re-examined the pattern of age-related pathology in the hermaphrodite gonad using Nomarski microscopy. Initially, shrivelling (atrophy) of the distal gonad was seen, which was followed by disintegration (fragmentation) in most animals by day 12 of adulthood. While little remained of the distal germline of such animals, atrophy of the proximal germline distal to the spermatheca often left one or more enlarged oocytes within the gonadal sheath.

To quantify germline degeneration, we used an approach based on an earlier study [20], defining 5 stages of gonad aging. At stage 1 the gonad was full sized and youthful in appearance; at stage 2 it was still intact, but showed slight signs of atrophy and deterioration; at stage 3 it showed clear atrophy and signs of impending disintegration (i.e. marked narrowing at one or more points along the gonad); and at stage 4, fragmentation had occurred (Figure 1B). At stage 5, which was occasionally seen, overall decrepitude was too severe to discern distal gonad fragments. Scoring germline disintegration on day 1, 8 and 12 of adulthood (20°C), and comparing these, confirmed that there is an age-related increase in gonad deterioration (Figure 1C). By day 8 more than a third of animals showed disintegrated gonads, rising to 80% by day 12.

Gonad degeneration was accompanied by a progressive reduction in germ cell number. Between day 4 and day 8, the number of germ cells decreased markedly (Figure 1D, 1E, measured by DAPI staining). Thus, between the end of reproduction (around day 4) and day 12 of adulthood, the aging germline undergoes severe atrophy in the main, syncytial component, and hypertrophy of cellularized oocytes (Figure 1A).

### Continued physiological apoptosis in the aging hermaphrodite germline

We then considered the causes of these age-related pathologies. Uterine tumor formation appears to result from run-on of growth in unfertilized oocytes [16–19]. We explored the possibility that distal gonad disintegration also results from run-on of functions that promote early life fitness.

In the distal arm, meiotic cells line the gonad as a syncytium and are connected to a central core of cytoplasm (or rachis). Production of cytoplasm by germ cells, and germ cell apoptosis, generates a distal to proximal flow of cytoplasm which provisions expanding oocytes near the bend in the gonad [21]. Such “physiological” apoptosis (PA), which is distinct from stress-induced apoptosis (e.g. it is independent of p53 [*cep-1*]), is a major cause of germline cell loss in early adulthood: at least 50% [22] and as many as 97% [23] of germline cells undergo PA. By provisioning developing oocytes, in a fashion analogous to nurse cells in mammals, PA contributes to reproductive fitness [24]. One possibility is that PA is not switched off in later life and that apoptotic run-on promotes atrophy. Plausibly, maintenance of the distal gonad reflects a balance between the rate of germ-cell proliferation and of PA. The number of mitotic germ cells does decline with age in hermaphrodites [18], which may contribute to senescent gonad atrophy. Here we explore the possibility that in the context of declining rate of germ-cell proliferation, run-on of PA promotes gonad degeneration.

**Figure 2:**
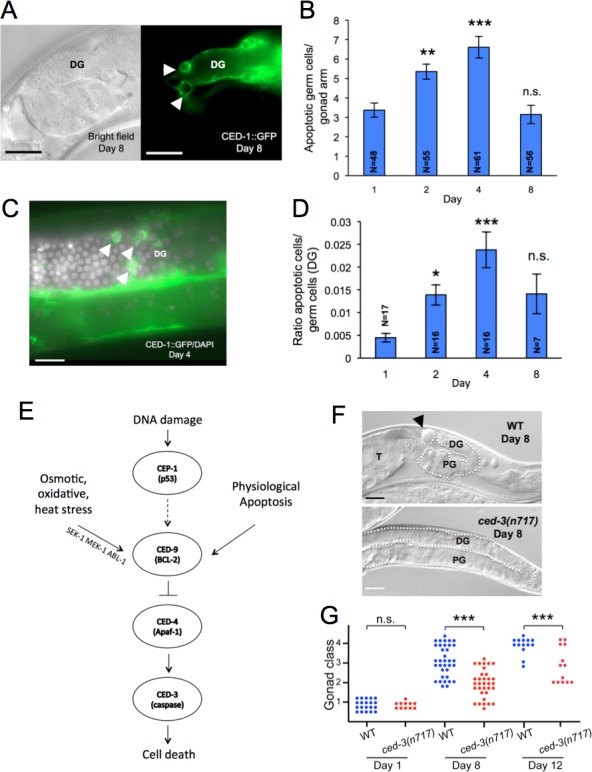
Post-reproductive run-on of germline apoptosis **A.** Presence of apoptotic cell corpses in later life. Apoptotic (CED-1::GFP positive) cells (arrowheads) in the germline of old hermaphrodite (day 8 of adulthood). **B.** Age changes in mean number of apoptotic cells/gonad arm. **C.** Overlaid images of DAPI-stained nuclei and CED-1::GFP positive apoptotic cells in post-reproductive hermaphrodite gonad. **D.** Number of apoptotic corpses normalized to nuclear number. **E.** Genetic pathways regulating cell death in *C. elegans* (simplified scheme). **F.**, **G.** *ced-3(n717)* suppresses gonad degeneration. In **F.** the wild-type (WT) worm exhibits gonad class 4 and the *ced-3* mutant gonad class 2; note in the latter that the gonad syncytium occupies much of the proximal gonad. **A.**, **C.**, **F.** Scale bar, 20 μm. D.G., distal gonad, P.G., proximal gonad. Error bars, S.E.M.; n.s., not statistically significant; * 0.01 < *p* < 0.05, ** 0.001 < *p* < 0.01, *** *p* < 0.001, Student's t test, compared to day 1 **B.**, **D.**, Wilcoxon-Mann Whitney test compared to wild type **G.**. N, sample size.

To test for the presence of such post-reproductive PA, we scored age changes in levels of apoptosis, using CED-1::GFP which is visible in somatic sheath cells that surround each apoptotic corpse [25]. This showed that apoptotic corpses are abundant until at least day 8 of adulthood (Figure 2A, 2B). Normalization to the number of germ cell nuclei revealed an increase in apoptotic corpse frequency from day 1 to day 4, followed by a modest decrease by day 8 (Figure 2C, 2D), consistent with previous observations [18]. We also scored age changes in cells taking up the vital dye SYTO 12, which confirmed the presence of apoptotic corpses up to at least day 8 (Supplementary Figure 1). These results imply that worms do not switch off PA after the cessation of reproduction, and suggest the presence of an apoptotic open faucet (i.e. run-on of PA) in the aging germline.

### Altered gonad degeneration rate in germline apoptosis mutants

**Figure 3:**
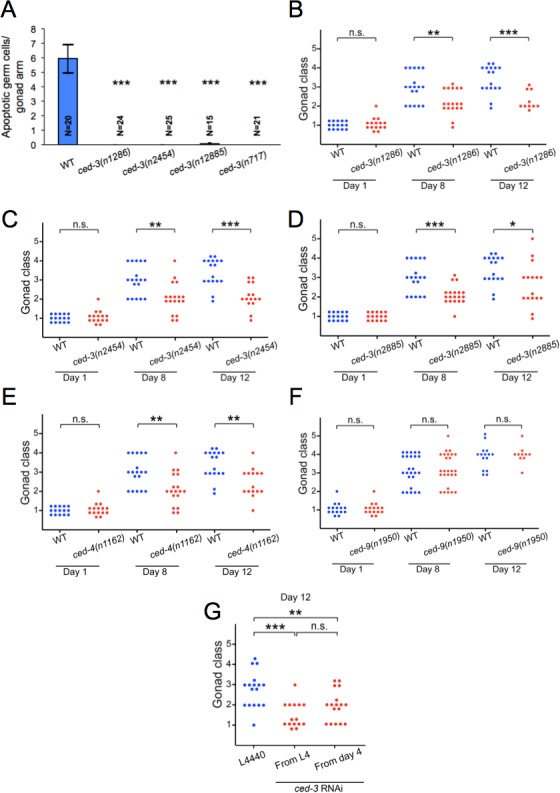
Suppression of gonad degeneration in apoptosis-defective mutants **A.** For all four *ced-3* alleles studied here it was confirmed using SYTO 12 staining that physiological germline apoptosis is suppressed. **B.**-**D.** *ced-3* suppresses gonad degeneration. **B.** *ced-3(n1286)*. **C.** *ced-3(n2454)*. **D.** *ced-3(n2885)*. **E.** *ced-4(n1162)*. There is no statistically significant difference between the magnitude of the effect of any of the five *ced* mutations on gonad disintegration on day 8 or 12, except between *ced-3(n717)* and *ced-3(n2454)* on day 12 (*p* = 0.04). **F.** *ced-9(n1950)* has no effect on gonad degeneration rate. **G.** N2 with *ced-3* RNAi from day 4 of adulthood onwards. * 0.01 < *p* < 0.05, *** *p* < 0.001, compared to wild type (Wilcoxon-Mann Whitney test); n.s., not statistically significant; error bars, S.E.M.; N, sample size.

To probe whether continued, post-reproductive apoptosis promotes hermaphrodite gonad degeneration, we blocked apoptosis using the mutation *ced-3(n717)* (cell death defective), which abrogates function of the executioner caspase CED-3 and blocks both somatic and germline apoptosis [22] (Figure 2E). This caused a marked inhibition (deceleration) of gonadal degeneration, though degeneration still did eventually occur (Figure 2F and 2G). Three other *ced-3* mutant alleles, *n1286, n2454* and *n2885*, like *n717*, similarly suppressed germline apoptosis (Figure 3A) and slowed gonad degeneration (Figure 3B-3D). *ced-4(n1162)* (Apaf-1-like protein), another mutation that inhibits somatic and germline apoptosis [22] also inhibited gonad degeneration (Figure 3E).

To exclude the possibility that this inhibition of gonad degeneration is caused by loss of somatic apoptosis, we tested the gain-of-function mutation *ced-9(n1950)* (BCL-2-like protein), which suppresses somatic apoptosis, but not physiological apoptosis in the germline [22] (Figure 2E). *ced-9(n1950)* did not suppress gonad degeneration (Figure 3F). Moreover, RNAi inhibition of *ced-3* function from day 4 of adulthood onwards was sufficient to inhibit gonad degeneration (Figure 3G).

Taken together these results support the hypothesis that germline apoptosis in later life promotes gonad atrophy. However, a caveat here is that as *ced-3* and *ced-4* mutants age, the distal gonad syncytium expands around the bend of the gonad arm, eventually filling most of the gonad distal to the spermatheca [22] (Figure 2F). Thus a formal possibility is that a mechanism other than run-on of PA causes distal gonad atrophy, and that atrophy can be suppressed by germline hypertrophy caused by blocking PA.

**Figure 4:**
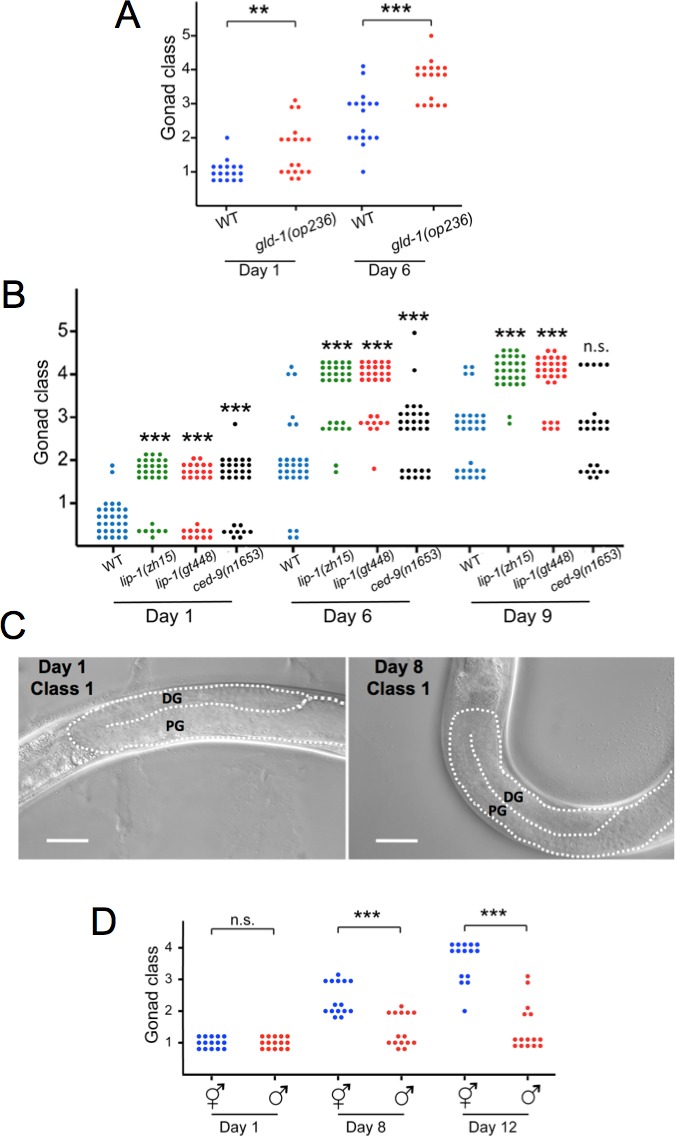
Germline apoptosis promotes gonad degeneration **A.** *gld-1(op236)* causes accelerated gonad degeneration. **B.** *lip-1(zh15)*, *lip-1(gt448)* and *ced-9(n1653)* cause accelerated gonad degeneration. Increased germline apoptosis is a temperature-sensitive phenotype, therefore trials were performed at 25°C, and effects on gonad degeneration scored on day 6 rather than day 8. **C.**, **D.** Comparison of hermaphrodite and male gonads in young and old adults. Scale bar, 20 μm; DG, distal gonad, PG, proximal gonad. n.s., not statistically significant; ** 0.001 < *p* < 0.01, *** *p* < 0.001, Wilcoxon-Mann Whitney test compared to wild type hermaphrodites.

To exclude this we took a different approach to test the PA open faucet hypothesis further, and asked whether increasing the level of germline apoptosis would increase the rate of gonad degeneration. Mutations in *gld-1* (defective in *g*erm *l*ine *d*evelopment) can have a variety of phenotypic effects, including formation of germline tumours and abolition of oogenesis [26]. *gld-1(op236)* is an unusual allele that increases sensitivity to damage-induced germline apoptosis, and causes a temperature-sensitive increase in PA; it also causes an extension in the pachytene region which reduces the number of oocytes in the proximal gonad arm [27]. At 25°C *gld-1(op236)* increased gonad degeneration rate (Figure 4A). We also tested three other mutants that increase frequency of germline apoptosis, *lip-1(zh15)*, *lip-1(gt448)* (*lip-1* = *l*ateral-signal-*i*nduced *p*hosphatase *1*) and *ced-9(n1653)* (25°C) [22, 27, 28]. Again, all increased gonad degeneration rate (Figure 4B). These results suggest that post-reproductive germline apoptosis is sufficient to promote gonad degeneration.

We examined in more detail the effects of *gld-1(op236)*, first confirming that it increases PA levels (Supplementary Figure 2B). To verify that acceleration of gonad disintegration by *gld-1(op236)* is caused by increased germline apoptosis, we examined a *gld-1(op236); ced-3(n717)* double mutant. This showed significantly reduced gonad disintegration relative to *gld-1* alone (Supplementary Figure 2A), although *gld-1* still slightly increased gonad degeneration in *ced-3* worms (*p* < 0.05). The increase in apoptosis rate in *gld-1(op236)* mutants is partially dependent upon CEP-1 (p53-like protein) [27]. We confirmed this dependency (Supplementary Figure 2B), but also found that effects of *gld-1* on gonad degeneration were not *cep-1* dependent (Supplementary Figure 2C). These results suggest that acceleration of gonad degeneration by *gld-1(op236)* is partially but not wholly attributable to increased PA, and that PA-dependent but not *cep-1*-dependent apoptosis promotes gonad degeneration.

Two forms of apoptosis can occur in the germline: physiological apoptosis (PA) and stress-induced apoptosis (SIA). Our working model is that post-reproductive PA, perhaps occurring due to run-on, causes gonad degeneration, but it remains possible that SIA plays a role. One possibility is that stress during normal aging induces SIA which promotes gonad disintegration. SIA can be induced by DNA damage *via* induction of EGL-1, CED-13 and CEP-1/p53, or by oxidative, osmotic or thermal stress *via* the MAPK kinases MEK-1 and SEK-1, and the p53 antagonist ABL-1 [29] (Figure 2E). If PA causes gonad degeneration, then neither *cep-1(*RNAi*)* nor the *abl-1(ok171)* mutant should suppress gonad degeneration, and this proved to be the case (Supplementary Figure 3A, 3B). We also disabled both mechanisms of SIA by subjecting *abl-1(ok171)* mutants to *cep-1*(RNAi) but still saw no alteration in rate of gonad degeneration (Supplementary Figure 3C). This argues against the idea that germline apoptosis in late life is promoted by aging-related stress.

Taken together these results are consistent with the view that continued PA in the germline contributes to age-related gonad degeneration. However, blocking PA delays gonad degeneration rather than blocking it entirely (Figure 2G, Figure 3), indicating the presence of additional pathogenetic mechanisms. A plausible additional mechanism is declining germ-cell proliferation rate.

### Gonad disintegration does not occur in males

In males germline apoptosis does not occur [22]. If post-reproductive PA contributes to gonad degeneration, then one would expect less germline degeneration in males. This proved to be the case. Only a slight deterioration in gonad appearance was observed in elderly males, and gonad disintegration was never seen (Figure 4C, 4D). Moreover, the minor signs of aging in the male gonad appeared unaffected by *ced-3(n717)* (Supplementary Figure 4).

To test whether the absence of gonad degeneration in males is wholly attributable to the absence of germline PA, we examined *fog-1(q253)* and *gld-1(q126)* mutant males, which are somatically male but exhibit germline apoptosis due to germline feminization [22, 26, 30]. However, age-related gonad disintegration was not seen in either mutant (Supplementary Figure 5).

These results identify a major sex difference in aging pathology in *C. elegans*: hermaphrodite-specific disintegration of the gonad. That switching on PA in the male germline is not sufficient to induce gonad disintegration could argue against a role for PA in gonad atrophy. Alternatively, this finding could instead reflect the importance of a balance between rates of germ-cell proliferation and PA in maintenance of gonad biomass, and relatively high germ-cell proliferation rates in older males. According to this model, run-on of PA only causes atrophy in the context of declining germ-cell proliferation rate.

### Life-extending effects of *ced-3* abrogation not detected

Does gonad pathology promote late-life mortality? Several studies suggest not: for example, lifespan was not extended either by blocking uterine tumor growth [19] or blocking apoptosis using *ced-3(n1286)* [20]. However, several studies have reported increases in lifespan resulting from loss of *ced-3* function, either using *ced-3(n717)* [31], like *ced-3(n1286)* a strong allele [32], or *ced-3* RNAi initiated at L4 [33], which suppresses only germline apoptosis. We tested both interventions, but no increase in lifespan was seen (Supplementary Figure 6A, 6B, Supplementary Table 1). This discrepancy could imply that effects of *ced-3* (and perhaps gonad disintegration) on age-related mortality vary according to minor differences in culture conditions. To aid future researchers who might wish to understand this discrepancy we have provided raw mortality data for these trials (Supplementary Dataset 1, 2). We conclude that neither apoptosis nor gonad disintegration contribute to mortality, at least under standard conditions as used in this laboratory.

### *daf-2* can reduce gonad disintegration independently of germline apoptosis

Mutation of the *daf-2* insulin/IGF-1 receptor increases *C. elegans* lifespan [34] and delays reproductive senescence [35, 36] and gonad degeneration [20, 37]. One possibility is that *daf-2(−)* suppresses gonad degeneration by reducing PA. *daf-2* mutants fall broadly into two classes: class 1, e.g. *daf-2(m577),* which are less pleiotropic, and class 2, e.g. *daf-2(e1370)*, which are more pleiotropic [38]. We found that levels of PA were unaffected by *daf-2(m577)* but reduced by *daf-2(e1370)* (Supplementary Figure 7A, 7B). The observed effect of *daf-2(e1370)* is consistent with a previous study [39], though another report described increased germline apoptosis in *daf-2(e1370)* mutants [40]. Other pathways have also been shown to influence *C. elegans* reproductive aging, particularly TGF-β Sma/Mab signalling [18, 41, 42].

To see whether mutation of *daf-2* can suppress gonad disintegration in the absence of any apparent reduction in PA, we examined *daf-2(m577)* mutants (25°C), and saw a deceleration of gonad disintegration (Supplementary Figure 7C). These results imply that *daf-2* mutant suppression of gonad disintegration involves a mechanism unrelated to PA, though reduction in PA could play a role in class 2 mutants such as *daf-2(e1370)*. One possibility is that this mechanism is related to the smaller age decrease in mitotic germ cell populations seen in *daf-2* mutants [18, 37].

Extension of lifespan in *daf-2* mutants is dependent on the *daf-16* FoxO transcription factor [34]. It was reported that *ced-3* mutants are resistant to several stressors, and that this stress resistance is *daf-16* dependent [31, 43]. This raises the possibility that suppression of gonad disintegration by *ced-3(−)* is due to DAF-16 activation rather than prevention of PA. We therefore tested whether suppression of gonad disintegration by *ced-3* was *daf-16* dependent, by subjecting *daf-16(mgDf50)* null mutants to *ced-3* RNAi. Although *daf-16(0)* did not appear to restore gonad disintegration, the analysis was complicated by morphological abnormalities in treated worms, including new degenerative changes, characterized by numerous large vacuoles, perhaps reflecting necrosis (Supplementary Figure 8).

### Hypertrophy of terminal oocytes during aging

**Figure 5:**
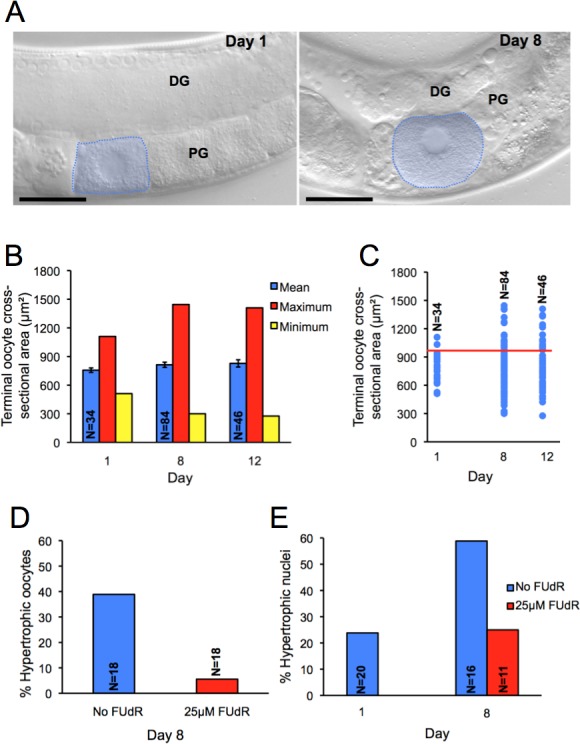
Appearance of hypertrophic terminal oocytes in aging *C. elegans* **A.** Normal terminal oocyte in young adult and hypertrophic un-ovulated oocyte in old adult. Scale bar, 20 μm; DG, distal gonad, PG, proximal gonad. **B.** Age change in size of oocytes; error bars, S.E.M. **C.** Age change in proportion of hypertrophic oocytes. Red line shows hypertrophy threshold. **D.** Effect of FUdR (25 μM) from day 1 on proportion of hypertrophic oocytes on day 8 of adulthood. **E.** Effect of FUdR (25 μM) from day 1 on proportion of hypertrophic nuclei on day 8 of adulthood. N, sample size.

Next, we characterized further the enlarged terminal oocytes seen in older worms. The terminal oocyte is the most proximal non-ovulated oocyte, closest to the spermatheca. In many worms a single enlarged oocyte was seen, often with several smaller oocytes on its distal side, all within the somatic gonadal sheath. In contrast to the decrepit tissues that surround them, such hypertrophic oocytes have a distinctly healthy, youthful appearance (Figure 5A). They also exhibit marked twitching movements, caused by spasmodic contractions of the gonadal sheath (Supplementary Videos 1, 2).

Measurement of terminal oocyte size (cross-sectional area) during aging revealed both an increase in maximum size (+30% on days 8 and 12 compared to day 1) and a decrease in minimum size, while mean size did not change (Figure 5B); thus, there is an age increase in terminal oocyte size variation. Defining oocyte hypertrophy as more than 1 standard deviation (S.D.) above the day 1 mean size (cross-sectional area > 945μm^2^), on days 1, 8 and 12, 6%, 27% and 33% of worms, respectively, contained hypertrophic un-ovulated oocytes (Figure 5C).

One possible cause of hypertrophy in terminal oocytes is endomitosis which, as previously noted, occurs in oocytes in post-reproductive hermaphrodites [44], and also in mutants defective in fertilization [45] or ovulation (Emo) [46]. Although oocyte hypertrophy was not noted in these prior studies, endomitosis could contribute to hypertrophy in later life, by mechanisms similar to those by which increased ploidy in hypodermal nuclei promote worm growth [47]. Consistent with this, we observed an increase in frequency of hypertrophic nuclei (> [mean size of day 1 nuclei] + 1 S.D., or > 27.2 μm^2^) in aging terminal oocytes (Figure 5E). Moreover, treatment from day 1 of adulthood with 5-fluoro-2′-deoxyuridine (FUdR, 25 μM), an inhibitor of DNA replication, markedly reduced the number of both hypertrophic oocytes (Figure 5D) and hypertrophic nuclei (Figure 5E). This is consistent with promotion by endomitotic run-on of hypertrophy in un-ovulated oocytes.

## DISCUSSION

**Figure 6:**
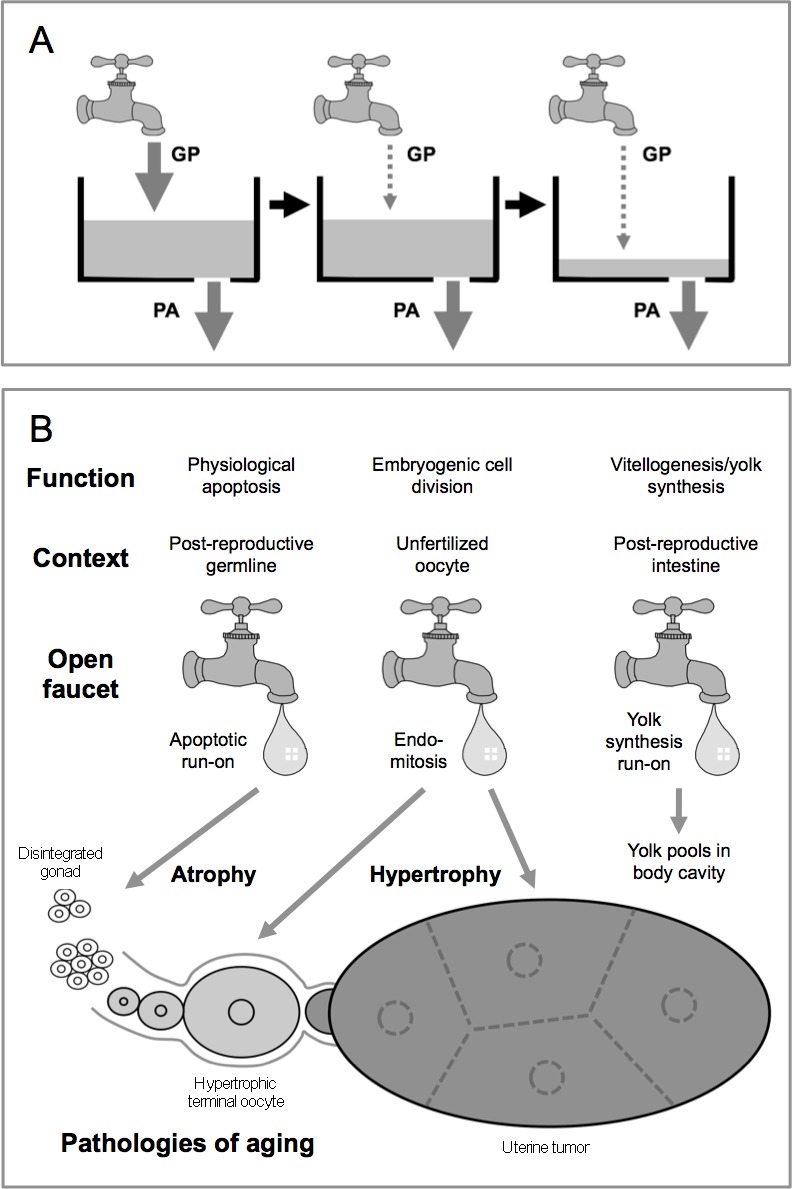
Role of hyperfunction in the generation of reproductive pathologies in *C. elegans* **A.** Faucet and sink model for the relationship between germ-cell proliferation (GP) and germline physiological apoptosis (PA) in the etiology of hermaphrodite gonad degeneration. Left: rate of GP and PA are equal, thereby maintaining gonad biomass. Center: A major decline in GP but not PA. Right: This leads to emptying of the sink (i.e. gonad atrophy). Possible scenarios whereby senescent gonad is prevented: in males, there is no PA, and GP rate is maintained; in *daf-2* mutants, GP rate is maintained. **B.** Role of open faucets in the origins of multiple senescent pathologies in the *C. elegans* hermaphrodite. Here several forms of run-on are operative. Apoptotic run-on promotes gonad disintegration (atrophy), while endomitotic run-on in unfertilized oocytes causes terminal oocyte hypertrophy and uterine tumors. Similarly, post-reproductive run-on of vitellogenesis by the intestine leads to yolk accumulation in the body cavity and elsewhere.

These results support the hypothesis that run-on of reproductive processes contribute to age-related pathogenesis in the *C. elegans* hermaphrodite gonad. We present evidence that post-reproductive physiological apoptosis promotes germline atrophy (Figure 6B), and also that run-on of endomitosis promotes hypertrophy in un-ovulated oocytes. The latter is consistent with previous evidence that endomitosis contributes to development of tumors from unfertilized oocytes in the uterus [19]. Moreover, post-reproductive hermaphrodites accumulate large quantities of yolk in the body cavity, as the result of run-on of intestinal yolk synthesis [48, 49] (Figure 6B).

### Post-reproductive physiological apoptosis and aging

Several observations support the view that post-reproductive physiological apoptosis in the germline promotes gonad disintegration: blocking germline but not somatic apoptosis delays degeneration; increasing germline apoptosis accelerates degeneration. This is reminiscent of the action of germline apoptosis as a driver of gonad atrophy during starvation-induced adult reproductive diapause in *C. elegans* [50], and also of follicular atresia in the loss of oocytes from the human ovary [51]. More broadly, these findings provide further illustration of how apoptosis can contribute to senescence, as recently reviewed [52].

In males, where PA does not occur, gonad disintegration does not occur either. However, inducing PA in the male gonad did not cause senescent atrophy (Supplementary Figure 5), suggesting that absence of major atrophy in wild-type males is not simply attributable to absence of PA. Instead, these findings support the view that run-on of PA leads to gonad degeneration only in the context of a decline in the germ-cell proliferation (GP) rate relative to that of PA (Figure 6A); the lack of atrophy in the male gonad suggests that male GP rate declines less with age than in hermaphrodites. By this view, declining GP and run-on of PA are primary and secondary causes of gonad degeneration. In *daf-2* mutants age-related loss of germline progenitor cells is reduced in *daf-2* mutants [18, 37]; thus, maintenance of GP production in later life could protect against gonad atrophy. The role of declining GP rate in gonadal senescence warrants further investigation. Our attempts to measure GP rate using 5-ethynyl-2′-deoxyuridine (EdU) labeling [53] were, unfortunately unsuccessful.

### Run-on rather than molecular damage as a possible cause of aging

These findings provide evidence that late life run-on of processes that promote fitness earlier in life contributes to senescent pathology in *C. elegans*. Thus, in at least some cases mechanisms promoting senescent pathology are distinct from molecular damage accumulation. In limited tests that we performed (Supporting Information, S9), no evidence of a role of molecular damage in the origins of the pathologies under scrutiny was detected. This is consistent with previous studies that have argued against the importance of reactive oxygen species in aging of *C. elegans* [54–56] and other organisms [6, 12]. However, a role for molecular damage in age-related pathology in the gonad cannot be ruled out. Consistent with the latter possibility, mutation of *ced-3* increases resistance to several stressors, particularly ER stress [31]. Also, molecular damage could contribute to age decline in GP rate.

### Understanding aging through senescent pathology rather than lifespan

Biogerontological studies of *C. elegans* usually use lifespan as a readout of aging. In this study we have instead focused on age-related pathology. The balance of evidence suggests that gonadal pathology (distal gonad involution, uterine tumors) have, at most, only a minor effect on lifespan. However, this does not mean that these pathologies are uninformative with respect to aging. Aging (senescence) manifests as set of pathologies, largely of endogenously origin, that increase in later life [1]. The key to understanding the biology of aging is to discover the proximate causes of age-related pathologies. Whether a particular pathology contributes to mortality will depend on context (species, culture conditions, the presence of other, more lethal pathologies). For example, aging promotes growth of tumors, which kill humans but not *C. elegans* [19]. Thus, studying gonad aging can define principles of age-related pathogenesis that lie at the heart of the aging process. In the present instance, we mainly focus on one likely driver of aging pathology in the gonad: post-reproductive physiological apoptosis in the germline, which is an example of hyperfunction [12].

## MATERIALS AND METHODS

### Culture conditions and strains

Maintenance of *C. elegans* strains was performed as previously described [57, 58]. Unless otherwise stated, worms were maintained at 20°C on NGM plates seeded with *E. coli* OP50. For experiments using male worms, L4 males were picked and maintained at low population density (5 worms per plate) to reduce life-shortening effects of culturing males in single-sex groups [59]. Strains used include N2 (wild type), AH102 *lip-1(zh15)* IV, AV106 *spo-11(ok79)* IV/*nT1 [unc-?(n754) let-?]* (IV;V), DR1563 *daf-2(e1370)* III, DR1567 *daf-2(m577)* III, GA184 *sod-2(gk257) I*, GA1801 *lip-1(gt448)* IV, GA1802 *ced-9(n1653ts)* III, GA1803 *gld-1(op236)* I*; ced-3(n717)* IV, GR1307 *daf-16(mgDf50)* I, JK560 *fog-1(q253)* I, JK4563 *gld-1(q126sd)* I*/hT2 [bli-4(e937) let-?(q782) qIs48]* (I;III), MD701 *bcIs39 [(lim-7) ced-1p::GFP + lin-15(+)]* V, MT1522 *ced-3(n717)* IV, MT2547 *ced-4(n1162)* III, MT3002 *ced-3(n1286)* IV, MT3970 *mab-5(mu14) ced-9(n1653)* III, MT4770 *ced-9(n1950)* III, MT8313 *ced-3(n2885)* IV, MT8354 *ced-3(n2454)* IV, TG34 *gld-1(op236)* I, and XR1 *abl-1(ok171)* X.

*fog-1(q253)* and *gld-1(q126)* males with feminized germlines were prepared by mating JK560 or JK4563 hermaphrodites with N2 males, then mating F1 males with JK560 or JK4563 hermaphrodites. From among the progeny, males were picked, and Fog males (homozygous for *fog-1(q253)* or *gld-1(q126)*) identified by the presence of oocytes in the gonad. *fog-1* males were aged at 25°C and *gld-1* males at 20°C.

### Microscopy

Worms were viewed on 2% agarose pads, using 0.2% levamisole as an anesthetic [60], typically at 400x magnification. Differential interference contrast (DIC) microscopy was performed using a Zeiss Axioskop2plus microscope connected to a Hamamatsu C10600 - Orca ER digital camera. The Volocity 5.2 (Improvision, Perkin Elmer) software was used for image acquisition and quantification.

### Germ cell quantification and apoptotic cell corpse assays

To quantify germ cell number worms were stained using 6′-diamino-2-phenylindole hydrochloride (DAPI) [50]. The number of apoptotic corpses in germlines was estimated using the vital dye SYTO 12 [22], or using CED-1::GFP [25].

To quantify germ cell number worms were stained using 6′-diamino-2-phenylidole hydrochloride (DAPI) as previously described [50]. Worms were fixed in ice-cold methanol for 5 min, washed with M9, and incubated in 500 ng/ml DAPI solution in the dark for 30 min. Worms were then washed once more with M9 and mounted on slides for imaging. DAPI-stained worms were imaged at 200x to obtain an image of the entire visible gonad arm. The open source Image J software (NIH image) with the ITCN plug-in was used to quantify the number of germ cells per gonad arm.

The number of apoptotic corpses in live worms was estimated using the vital dye SYTO 12 (Molecular Probes), as previously described [22]. Worms were incubated in the dark in 33 μM SYTO 12 for 4 hrs. Worms were then transferred to a freshly seeded OP50 plate for an hour for the stain to be expelled from the gut. Worms were placed on slides and SYTO 12-positive cells, viewed at 200x magnification, were counted manually. Alternatively, worms containing CED-1::GFP which is expressed in the gonad sheath was also used to count engulfed apoptotic cells [25].

### Gonad disintegration measurements

Gonad pathology was scored using an approach based on that previously described [20]. Gonad health status was categorized into five classes. Class 1: gonad full-sized and youthful in appearance. Class 2: gonad showing slight signs of atrophy and deterioration. Class 3: gonad showing clear reduction in diameter, and signs of impending fragmentation. Class 4: fragmented gonads. Class 5: gonad remnants barely recognisable or not discernible; this usually applied to non-motile, elderly worms that were close to death. DIC images of one gonad arm per worm were obtained, and given a score of 1-5 by three scorers who had no knowledge of the age or genotype of the worms. The rounded mean value of these three scores was assigned to each image.

To ensure an unbiased assessment of images of aging gonads, blind scoring was performed. To this end, all images were renamed and placed in random order (shuffled) before scoring. For such shuffling it was important both that the filename gave no clue about the origin of the file, and that the original image filename could be accurately restored after scoring (unshuffled). Such shuffling and unshuffling can be achieved manually, but to remove potential human error and speed things up, we developed a Python script that automates image shuffling and unshuffling. This script scans a folder for the images it contains and renames the images randomly to numbers 0 to [N-1], where N is the number of images in the folder. The script automatically creates a ‘key.csv’ file which lists the filenames of the shuffled images together with their original filenames. This file is then used by the inverse function of the script to restore the original filenames after the scoring has taken place.

Without further assistance the user could conduct blind scoring on the images, but they still would have to revert the scoring by hand by using the ‘key.csv’ file. We therefore automated this process as well, by generating an Excel spreadsheet during the shuffle phase with the first row listing the anonymized filename. In the subsequent rows the investigator can fill in any scoring/assessment results for each of these images. Using the ‘key.csv’ file the script will then also automatically unshuffle this spreadsheet, substituting the anonymized filenames with the original ones.

The software can run on Windows (PC), OSX, and Linux and is available as a zip file as Supplemental Resource 1 (image-shuffle-master). It is also available at https://github.com/groakat/image-shuffle.

### RNAi treatment

The RNAi clones *ced-3* and *cep-1* were obtained from the Ahringer library, and identity of their inserts confirmed by sequencing. RNAi by feeding was performed as previously described [61]. Unless otherwise stated, worms were raised on *E. coli* HT115 containing the empty vector L4440 (control RNAi) until the L4 stage and then transferred to plates seeded with the RNAi clone of interest.

### Survival analysis

Lifespan measurement trials were conducted much as previously described [38]. Briefly, worms were raised to L4 on control RNAi (20°C), and then transferred to plates seeded with *ced-3* RNAi or control RNAi (L4440 vector control). Worms were transferred to fresh plates daily during the reproductive period. Deaths were scored and worms that died from desiccation, rupture or bagging were censored.

### Statistical comparisons

The non-parametric Wilcoxon-Mann Whitney test was used in experiments measuring gonad ageing. Data obtained as a mean value for a group was analysed using the pair-wise Student's t test. The Tukey Kramer test was used to adjust for multiple comparisons. Lifespan datasets were compared using the non-parametric log rank test, using JMP software (SAS Institute).

## SUPPLEMENTARY MATERIAL FIGURES AND TABLE










