# Supplementary material for: Run-on of germline apoptosis promotes gonad senescence in C. elegans
Source: Oncotarget. 2016 May 31;7(26):39082–96. doi: 10.18632/oncotarget.9681 (PMC5129915; doi:10.18632/oncotarget.9681)
Supplement: Supplementary file 1 [file oncotarget-07-39082-s001.pdf]

Run-on of germline apoptosis promotes gonad senescence in *C. elegans*

Supplementary Material
List of contents

Supplementary Figures 1-9. See below.

Supplementary Table 1. Absence of life-extending effects of loss of *ced-3* function. Below Supplementary Figure 6.

Supplementary Video 1, 2. Spasmodic contractions of gonadal sheath cause terminal oocyte motility in elderly hermaphrodites. These show two characteristic examples of motility in hypertrophic terminal oocytes, apparently resulting from contractions of the gonadal sheath. 9 day old worms (20˚C, on *E. coli* OP50). In both videos the anterior of the worm is to the left, and one or more smaller unfertilized oocytes are distal (to the right) of the terminal oocyte. Above the terminal oocyte, remnants of the distal gonad arm are visible. To the left of the terminal oocyte is the residual spermatheca and beyond that, to the far left, is the distal end of a uterine tumor.

Supplementary Datasets 1, 2. Raw mortality data for *ced-3* survival experiments. The data table format is designed (by M. Ziehm) to maximise data transparency and experimental reproducibility. Data includes specification of causes of data censoring, as follows. bag = bag of worms (death from internal hatching of eggs); cont = contaminated (bacterial or fungal infection); dis = disappeared (investigator failed to locate worm); kil = killed (worm accidentally killed during handling); lst = lost (censored without a recorded reason); otw = on the wall (worms climbed wall of Petri dish and died from desiccation); rup = rupture (internal organs extensively extruded through vulva). Supplementary Dataset 1 corresponds to both Supplementary Figure 6A and A in Supplementary Table 1, and Supplementary Dataset 2 to both Supplementary Figure 6B, and B in Supplementary Table 1.

Supplementary Resource 1. Image shuffle software. Available at https://github.com/groakat/image-shuffle

Supplemental results

Little effect of molecular damage on age-related gonad pathology
If aging is caused by stochastic molecular damage, then induction of increased levels of oxidative damage should accelerate the development of gonad pathology. High levels of pro-oxidants can increase nematode mortality [1, 2], but this could reflect toxic effects rather than acceleration of aging; if gonadal aging rate is a function of molecular damage accumulation, then even modest increases in molecular damage levels should be sufficient to accelerate the development of this pathology. We therefore tested effects on gonadal aging of two interventions previously shown to increase both protein oxidative damage and sensitivity to oxidative stress, but not to reduce lifespan: the *sod-2(gk257)* mutation

(mitochondrial superoxide dismutase), and iron supplementation (9 mM ferric ammonium citrate) [2-4]. Neither treatment accelerated gonad degeneration (Supplemental Figure 9A-D).

Programmed molecular damage occurs in the germline in the form of DNA breaks generated to initiate recombination during meiosis. We examined *spo-11(ok79)* mutants, which are unable to generate DNA breaks during meiosis, and therefore have reduced levels of DNA damage in the germline [5], but no effect on gonad degeneration was seen (Supplemental Figure 9E). These findings argue against a role of molecular damage in gonad degeneration, although they do not exclude it.

Supplementary methods

Treatment with iron

To induce oxidative damage, worms were raised at 20°C on *E. coli* OP50 seeded NGM plates until L4 stage, and then transferred to 9mM ferric ammonium [iron (III)] citrate (FAC) NGM plates for the remainder of the experiment [2]. NGM FAC plates were kept away from light to avoid reduction of iron (III) to iron (II).

Oxidative damage assays

Day 1 adult worms were rinsed off NGM plates, washed with M9 to remove bacteria and stored at -80˚C. Samples were thawed, centrifuged and supernatant was removed. 100 µl of CellLytic (Sigma) lysis buffer with 1X protease inhibitor (Roche) was added to samples and they were sonicated 3 times for 30 seconds using a Bioruptor (Cosmo Bio). Samples were then centrifuged for 30 min at 20,000 rpm at 4˚C to remove debris. Protein concentration was determined in the supernatant for each sample using the Bradford assay (Bio-Rad). Each sample was normalized to 20 µg in a total volume of 5 µl. 5 µl of 12% SDS and 10 µl of a 1:10 dilution of dinitro-phenylhydrazine (DNPH) was added to samples, which were incubated at room temperature for 15 minutes. Denatured protein samples were then separated on an SDS-PAGE gel and transferred to a nitrocellulose membrane. Protein carbonylation was detected by anti-dinitrophenol antibodies (Millipore). ECL Plus Western Blotting Detection System (Amersham Biosciences) was used to detect binding of the antibody as recommended by the manufacturer. A sheet of Hyperfilm (Amersham Biosciences) was placed on the membrane inside the developing cassette using a Kodak X-Omat developer. Films were scanned and density of the entire lane or individual bands were quantified using ImageQuant TL (GE Healthcare Europe).

Supplementary References

1. Adachi H, Fujiwara Y and Ishii N. Effects of oxygen on protein carbonyl and aging in *Caenorhabditis elegans* mutants with long (*age-1*) and short (*mev-1*) life spans. J Geront. 1998; 53A:B240-B244.
2. Valentini S, Cabreiro F, Ackerman D, Alam MM, Kunze MBA, Kay CWM and Gems D. Manipulation of *in vivo* iron levels can alter resistance to oxidative stress without affecting ageing in the nematode *C. elegans*. Mech Ageing Dev. 2012; 133:282-290.
3. Doonan R, McElwee JJ, Matthijssens F, Walker GA, Houthoofd K, Back P, Matcheski A, Vanfleteren JR and Gems D. Against the oxidative damage theory:

Superoxide dismutases protect against oxidative stress but have little or no effect on lifespan in C. elegans. Genes Develop. 2008; 22:3236-3241.
4. Van Raamsdonk JM and Hekimi S. Deletion of the mitochondrial superoxide dismutase sod-2 extends lifespan in Caenorhabditis elegans. PLoS Genet. 2009; 5(2):e1000361.
5. Dernburg AF, McDonald K, Moulder G, Barstead R, Dresser M and Villeneuve AM. Meiotic recombination in C. elegans initiates by a conserved mechanism and is dispensable for homologous chromosome synapsis. Cell. 1998; 94:387-398.
6. Gartner A, Boag PR and Blackwell TK. Germline survival and apoptosis. WormBook. 2008:1-20.
7. Barton MK and Kimble J. fog-1, a regulatory gene required for specification of spermatogenesis in the germ line of Caenorhabditis elegans. Genetics. 1990; 125:29-39.
8. Francis R, Barton MK, Kimble J and Schedl T. gld-1, a tumor suppressor gene required for oocyte development in Caenorhabditis elegans. Genetics. 1995; 139:579-606.

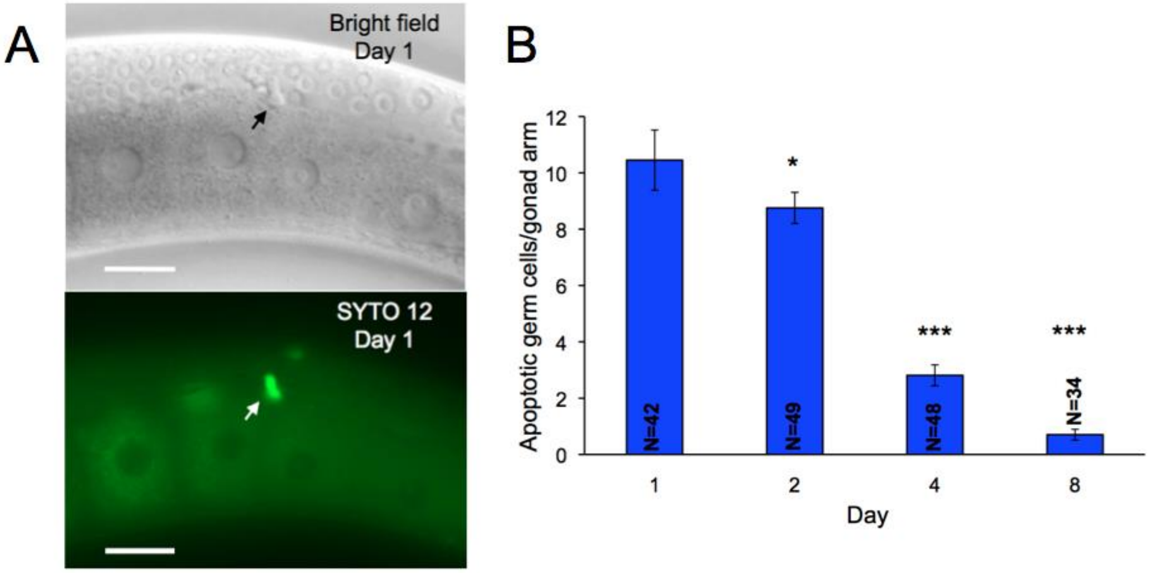

Supplementary Figure 1. Continued apoptosis in late life in the hermaphrodite germline. **A.** SYTO 12 stained apoptotic cells in young adult hermaphrodite gonad. Arrowhead, apoptotic cells; scale bar, 20 µm. **B.** Numbers of apoptotic cells in wild-type adult hermaphrodites of different ages (20˚C). * $0.01 < p < 0.05$, *** $p < 0.001$, Student's t test, compared to day 1; error bars, S.E.M.. N, sample size. Note that SYTO 12 staining gives a lower estimate of numbers of apoptotic cells than CED-1::GFP. This could reflect greater sensitivity of CED-1::GFP at identifying cells in the early stages of apoptosis [6].

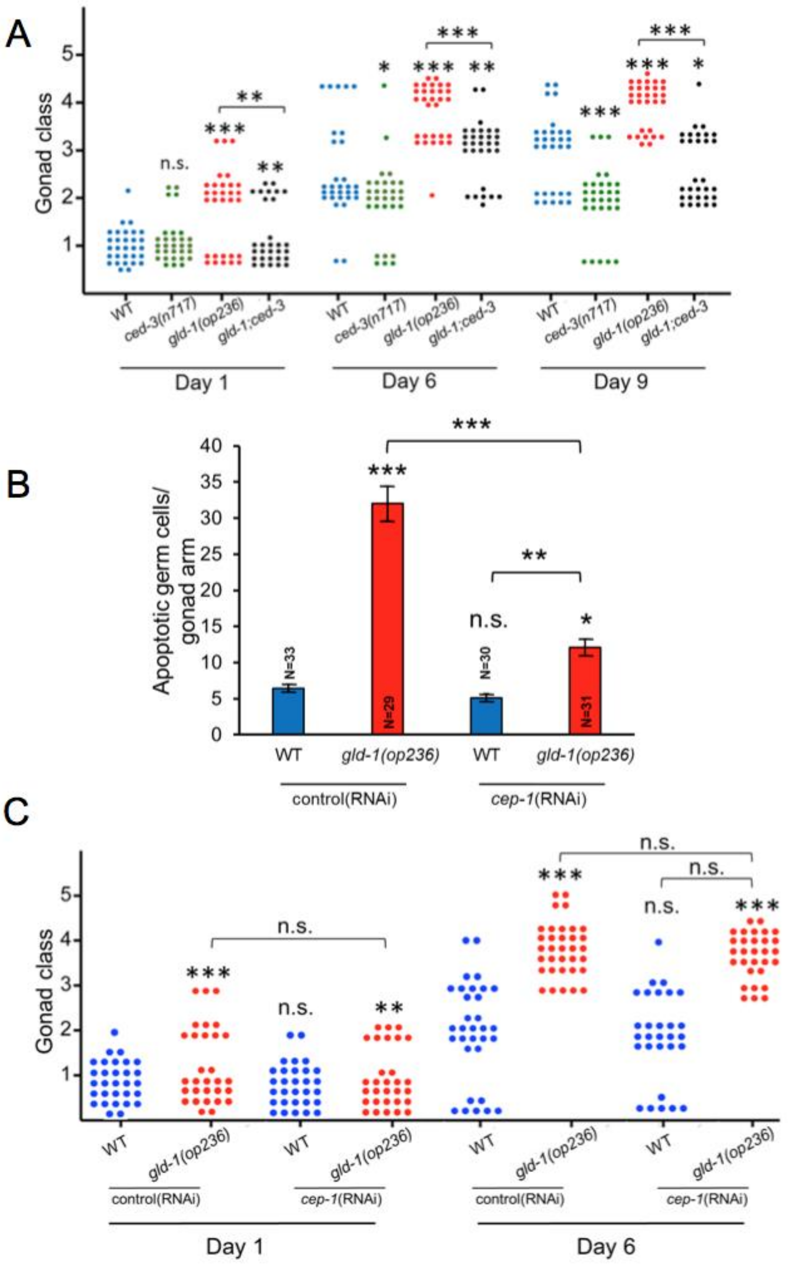

Supplementary Figure 2. Effects of *gld-1(op236)* on gonad disintegration are dependent upon *ced-3* but not *cep-1*.

A. Acceleration of gonad disintegration by *gld-1(op236)* requires apoptosis. **B.** Increased germline apoptosis in *gld-1(op236)* mutants is partially *cep-1* dependent. **C.** Increase in gonad disintegration in *gld-1(op236)* mutants is not *cep-1* dependent. Trials conducted at 25˚C. Asterisks not atop pairing bars denote comparison to wild type (WT) control. Statistical comparisons were performed using the Wilcoxon-Mann Whitney test (**A.**, **C.**), or the Tukey Kramer test (**B.**). * 0.05 > P > 0.01; ** 0.01 > P > 0.001; *** <0.001. Error bars, S.E.M.; n.s., not statistically significant.

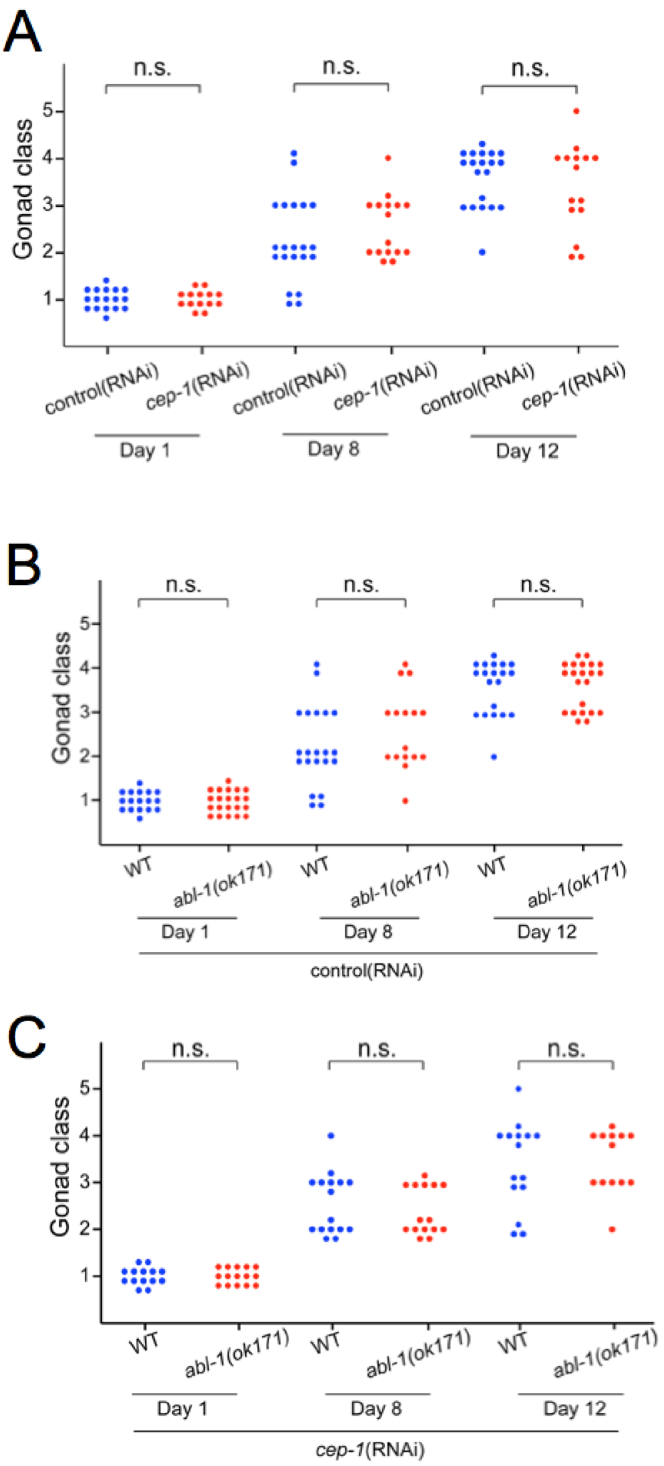

Supplementary Figure 3. Effects on gonad disintegration of genes required for stress-induced apoptosis (SIA) in the germline. **A.** *cep-1* (RNAi), **B.** *abl-1(ok171)*, **C.** *abl-1(ok171) cep-1(RNAi)*. n.s., not statistically significant, compared to wild type, Wilcoxon-Mann Whitney test.

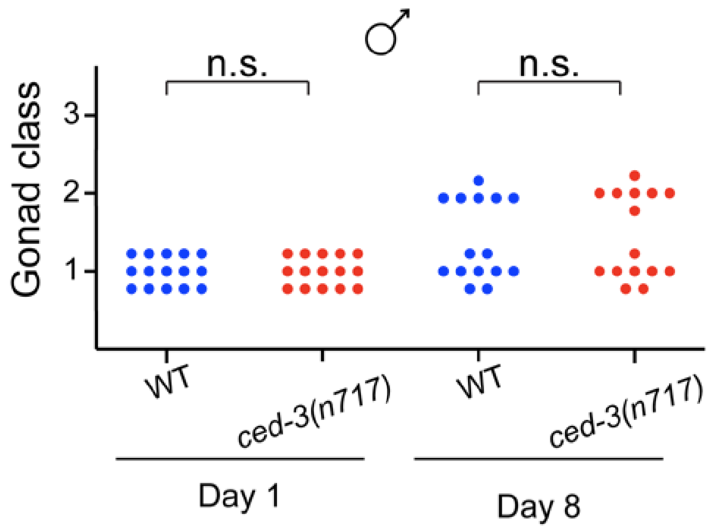

Supplementary Figure 4. No effect of *ced-3(n717)* on male gonad pathology. n.s., not statistically significant, compared to wild type, Wilcoxon-Mann Whitney test.

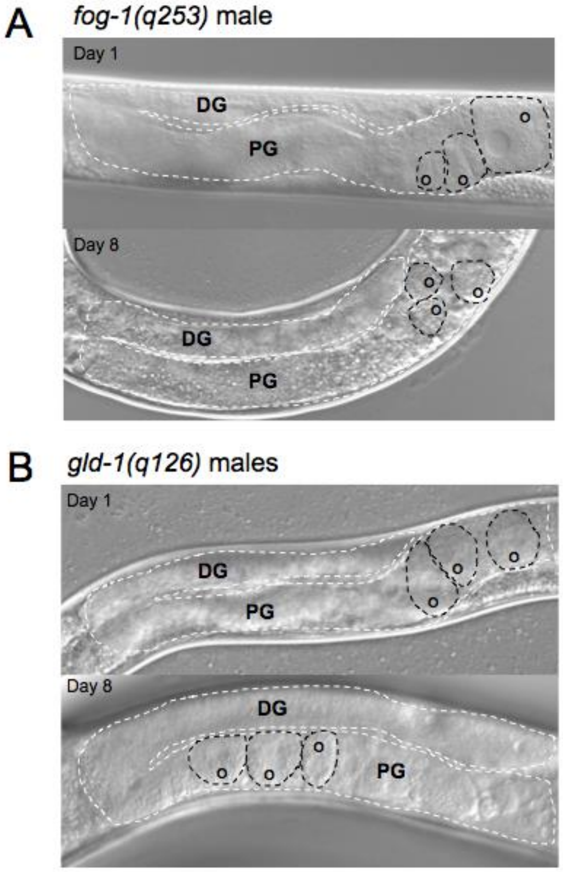

Supplementary Figure 5. Germline feminization does not lead to gonadal disintegration in aging males. A. *fog-1(q253)*. **B.** *gld-1(q126)*. The *fog-1* (*f*eminization *o*f the *g*erm line) mutation causes male to female sexual transformation of germline tissue without apparently altering somatic tissues [7]. The *gld-1(q126)* (defective in *g*erm *l*ine *d*evelopment) mutation is a class D (Fog) allele [8]. DG, distal gonad; PG, proximal gonad; o, oocyte.

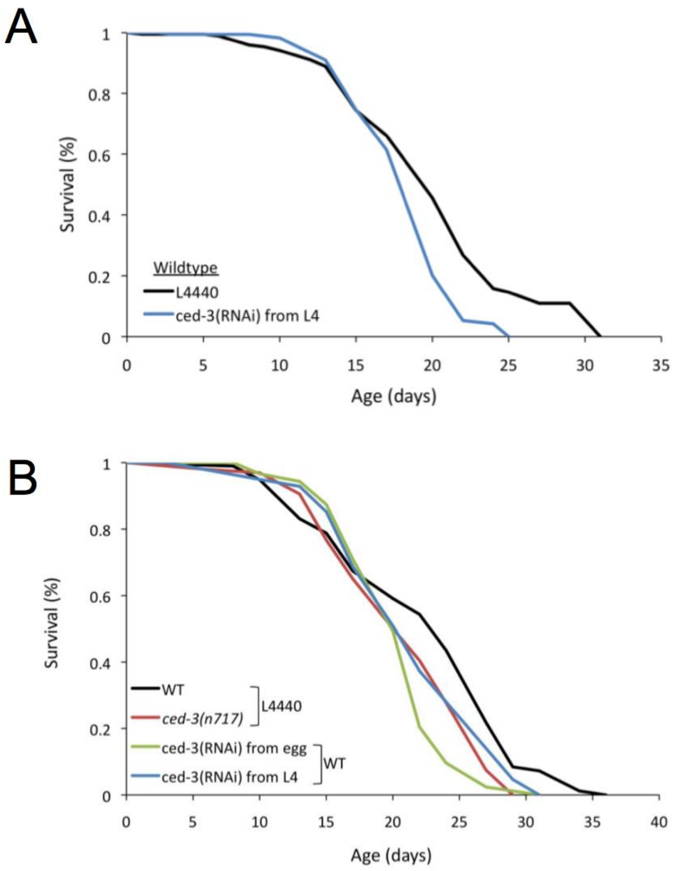

Supplementary Figure 6. Absence of life extending effects of loss of *ced-3* function. A. RNAi of *ced-3* initiated at L4 stage. **B.** *ced-3(m717)* or *ced-3* RNAi from egg or L4 stage.

Supplementary Table 1. Absence of life-extending effects of loss of *ced-3* function

Treatment	Mean lifespan [Median] (days)	% of control lifespan[1]	*p* vs. control (log rank)	Deaths [censored]
**A**				
N2, L4440 (RNAi vector control)	19.4 [20]			63 [37]
N2, *ced-3*(RNAi)	18.0 [20]	-8% [0]	0.0001	88 [12]
**B**				
N2, L4440 (RNAi vector control)	22.5 [24]			86 [11]
ced-3(n717), L4440	20.9 [22]	-7% [-8%]	0.008	94 [5]
N2, *ced-3*(RNAi) from egg	21.4 [22]	-5% [-8%]	0.0001	84 [16]
N2, *ced-3*(RNAi) from L4	20.4 [20]	-9% [-17%]	0.06	89 [11]

[1]Percentage of mean lifespan [of median lifespan]. Assays performed at 20˚C.

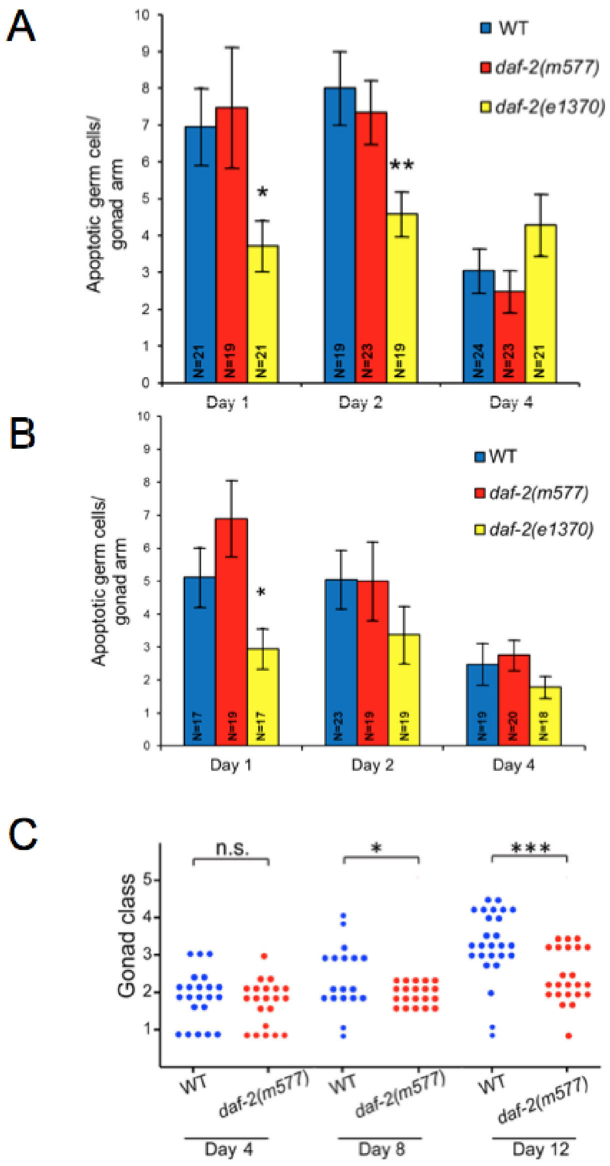

Supplementary Figure 7. Evidence that suppression of gonad disintegration by *daf-2* is not caused by reduced PA. A., B. The number of SYTO 12 staining cells per gonad arm in *daf-2(e1370)* and *daf-2(m577)* mutants. **A.**, 20°C; **B.** 25°C. Comparison to age-matched control (N2), Student's t test. **C.** Inhibition of gonad disintegration by *daf-2(m577)*, 25°C. Comparison to age-matched control (N2), Wilcoxon-Mann Whitney test. * 0.05 > P > 0.01; ** 0.01 > P > 0.001; *** <0.001. Error bars, S.E.M.; n.s., not statistically significant.

daf-16(mgDf50); ced-3 RNAi (day 12)

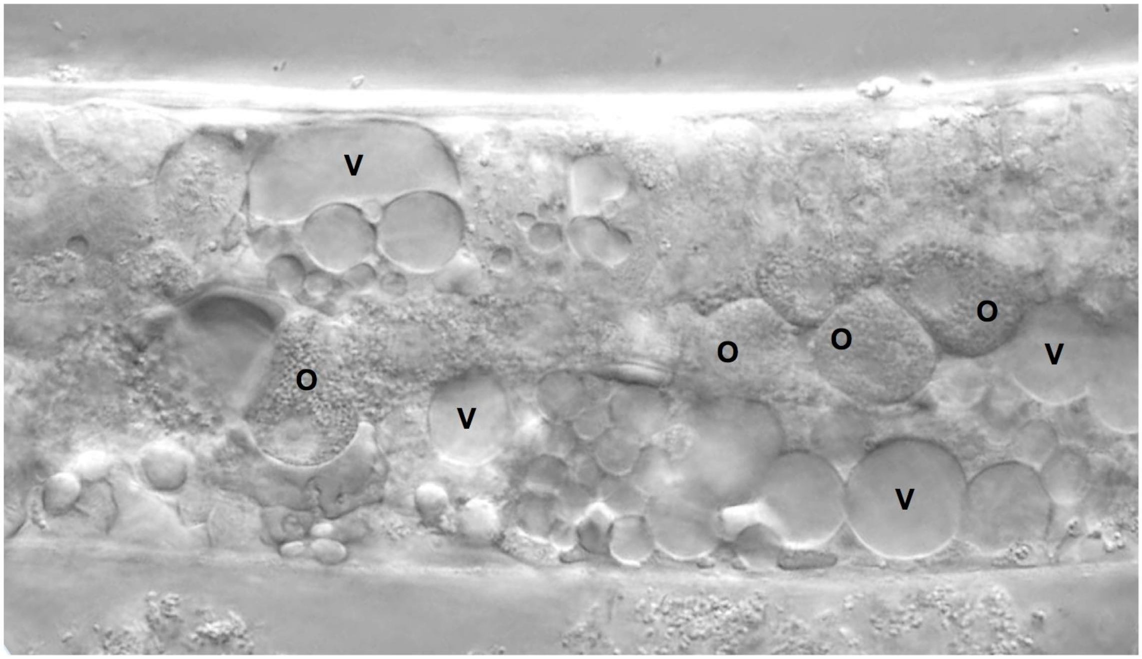

Supplementary Figure 8. Combined loss of *ced-3* and *daf-16* cause new pattern of gonad degeneration. 12 day old hermaphrodite of strain GR1307 *daf-16(mgDf50)*, subjected from L4 stage onward to *ced-3* RNAi (20˚C). Gonads show little atrophy but instead a new pattern of degeneration. V: Selected abnormal vesicles (V), possibly indicating cellular necrosis, and oocytes (O) are marked.

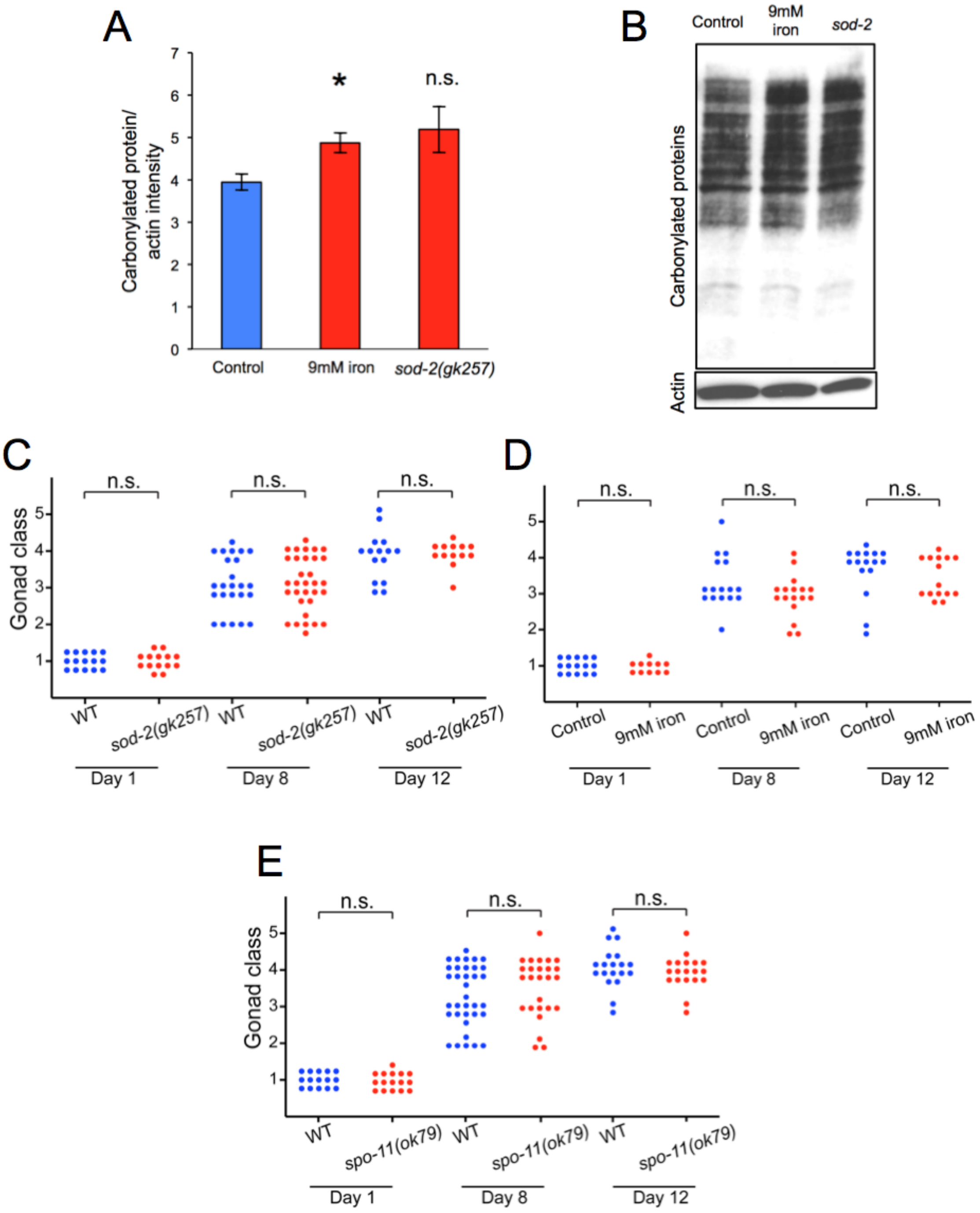

Supplementary Figure 9. Gonadal degeneration in hermaphrodites with altered levels of molecular damage. A., **B.** Effects of *sod-2(gk257)* and 9mM iron (ferric ammonium citrate, FAC) on protein carbonyl levels. **A.** Oxidized protein levels, normalized to actin; * $0.01 < p < 0.05$, Students t test, compared to control; error bars, S.E.M.; n.s., not statistically significant. **B.** Example of oxyblot showing protein oxidation. **C.**, **D.** No effect of **C.** *sod-2(gk257)* or **D.** 9 mM iron on rate of gonad degeneration. **E.** No effect of *spo-11(ok79)* on rate of gonad degeneration. n.s., not statistically significant, Wilcoxon-Mann Whitney test.
